# Association of L-arginine with heparin on the sperm capacitation improves *in vitro* embryo production in bovine

**DOI:** 10.21451/1984-3143-AR2019-0022

**Published:** 2019-11-18

**Authors:** Gester Breda Aguiar, Maria Clara Caldas-Bussiere, Valter Luiz Maciel, Carla Sobrinho Paes de Carvalho, Cláudio Luiz Melo de Souza

**Affiliations:** 1 Universidade Estadual do Norte Fluminense Darcy Ribeiro – UENF, Laboratório de Reprodução e Melhoramento Genético Animal, Campos dos Goytacazes, RJ, Brasil; 2 Universidade Estadual do Norte Fluminense Darcy Ribeiro – UENF, Laboratório de Engenharia Agrícola, Campos dos Goytacazes, RJ, Brasil

**Keywords:** *in vitro* fertilization, nitric oxide, blastocyst, membrane integrity, cattle

## Abstract

We aimed to evaluate the effects of L-arginine (L-arg) in the quality of *in vitro* heparin-induced capacitation of cryopreserved bovine spermatozoa and its effects on IVP. The experimental groups were: Control 0 hour without pre-capacitation, and groups of sperm capacitated for 30 min in the absence of COC with heparin (Control 30 min), with 1 mM L-arg and with 1 mM L-arg + heparin. The capacitation pattern was evaluated by chlortetracycline assay and the integrity of the plasma membrane (PM) and acrosome membrane (AM) by the association of Hoescht 33342 and propidium iodide. Further, we assess the sperm quality by the rate of *in vitro* blastocysts production. Treatment with 1 mM L-arg + heparin increased the percentage of capacitated sperm when compared to Control 0 hour and the treatment with heparin (61.1 *vs.* 18.2 and 47.0%, respectively, P<0.05). The addition of 1 mM L-arg to the medium has capacitated the spermatozoa (26.2 ± 3.8) but was less effective than heparin (47.0 ± 4.0) (P<0.05). There was no difference in the percentage of sperm with intact PM between treatments when compared to Control 0 hour (P>0.05). The group capacitated with 1 mM L-arg + heparin for 30 min increased the blastocyst rate compared to Control IVF (53.7 *vs.* 40.8%, P<0.05). We conclude that the addition of L-arg with heparin increases the number of capacitated spermatozoa *in vitro* with 30 min of pre-incubation in the absence of COC not altering the integrity of plasma and acrosomal membrane. This treatment in the absence of COC was the most effective method for blastocysts production, and the method of pre-incubation could be used to assess the role of other substances in the sperm capacitation and its effect on IVP.

## Introduction

Sperm capacitation process can be defined as the biochemical, biophysical, molecular, and metabolic changes of the sperm cell that confers the ability to fertilize an oocyte *in vivo* or *in vitro* (Bailey, 2010). In bovines, heparin, a glycosaminoglycan present in the female reproductive tract, has been considered the primary substance that promotes sperm capacitation *in vitro* (Parrish et al., 1988; Chamberland et al., 2001; Parrish, 2014). Through the penetration test on denuded homologous oocytes, the absence of heparin after 4 hours of culture demonstrated that only 31% of bovine spermatozoa could capacitate (Paes de Carvalho et al., 2003).

For improving the *in vitro* sperm capacitation, various substances have been used together with the fertilization media, such as L-arginine (L-arg) (O’Flaherty et al., 2004; Rodriguez et al., 2005; Roy and Atreja, 2008). L-arg plays a significant role in sperm motility, capacitation process, and induces acrosome reaction in cattle (O’Flaherty et al., 2004; Leal et al., 2009; Silva et al., 2014). Such effects have been linked to the synthesis of nitric oxide (NO) from L-arg by the sperm cell (O’Flaherty et al., 2004; Leal et al., 2009). In a previous study of our group, we demonstrated that the concentration of 1 mM L-arg + heparin was the most effective to improve sperm capacitation after 4 hours of incubation using cryopreserved sperm (Leal et al., 2012).

Nitric oxide is a reactive nitrogen species (RNS) that can act in inter and intracellular signaling, and as an antioxidant or free radical (Dixit and Parvizi, 2001). Nitric oxide is synthesized during the conversion of L-arg into L-citrulline in oxidative reactions. Although L-arg has been widely used in sperm capacitation (Funahashi, 2002; O’Flaherty et al., 2004; Leal et al., 2009), most of the studies do not evaluate its effect after capacitation, such as the *in vitro* embryo production. In a recent publication, L-arg improved embryonic developmental rates in cattle when added to the medium of IVF (Santana et al., 2016). Although common IVF medium is enriched to stimulate sperm capacitation, in this model, L-arg could influence both sperm and COCs.

Hence, we aimed to evaluate the effects of L-arg in the quality of *in vitro* heparin-induced capacitation of cryopreserved bovine spermatozoa and its effects on IVP to use L-arg on *in vitro* heparin-induced sperm capacitation of cattle and further evaluate its effects on the *in vitro* embryo production. For this, we investigated the plasma membrane integrity and the capacitation status of the sperm and we assess the sperm quality by the rate of blastocysts produced *in vitro*. With this, we hope to contribute in the study of the role of NO during bovine sperm capacitation and its impact on the *in vitro* produced embryos.

## Materials and methods

### Culture media

All reagents used in these experiments were obtained from Sigma-Aldrich Brasil Ltda (São Paulo, SP, Brazil) unless otherwise indicated. The media used in the process of *in vitro* embryo production were obtained from Progest Biotecnologia em Reprodução Animal (Botucatu, SP, Brazil).

### Sperm preparation and selection

The mini Percoll gradient was used for separation of viable spermatozoa after being thawed. For the 90% fraction, we used 540 µL of commercial Percoll (Amersham Pharmacia Biotech Ltda, Little Chalfont, UK) and 60 µL of 10× TALP. The fraction 45% was prepared by adding 200 µL of 90% Percoll and 200 µL of TALP Chamberland with 1% PVA (Chamberland et al., 2001). For the preparation of the mini Percoll gradient, the fraction of 45% was gently placed on the fraction of 90% in a 1.5 mL microtube.

Cryopreserved sperm were thawed and subjected to centrifugation at 700 × *g* for 5 min on mini Percoll gradient 45/90% (Eid et al., 1994). After centrifugation, the pellet was washed for 3 min (150 × *g*) in 400 µL of TALP Chamberland. The sperm motility and vigor were evaluated for quality confirmation, and then the sperm concentration was determined using a Neubauer chamber. The selected sperm were transferred to the respective capacitation tubes.

### Sperm capacitation

The medium used in the capacitation was modified Tyrodes (Parrish et al., 1988) supplemented with 6 mg/mL BSA, fatty acids free, 100 IU/mL penicillin and 100 µg/mL streptomycin, and containing 20 µg/mL heparin (except TL-arg), with or without 1 mM L-arginine (Leal et al., 2012) in an incubator at 38.5°C in humidified atmosphere of 95% air and 5% CO_2_. Sperm concentration was adjusted to 10 × 10^6^ sperm/mL for sperm evaluations and was adjusted to 2 × 10^6^ sperm/mL for *in vitro* fertilization.

The sperm capacitation was induced in 30 min of cultivation in the absence of COC for both experiments. In Experiment 1, we evaluated the acrosome and plasma membrane sperm integrity and the percentage of capacitated sperm. In Experiment 2, we tested the treatments (T-Hep, TL-arg, and TL-arg + Hep) on IVF to evaluate the rate of *in vitro* blastocysts production. The sperm capacitation in the presence of COC was only induced in the Control group with heparin (Control IVF).

### Simultaneous assessment of plasma membrane integrity

After the capacitation in absence of COC for 30 min and in the Control group 0 hour, an aliquot of 50 µL of sperm (25 × 10^6^ sperm/mL) was exposed to the Hoechst 33342 (40 mg/mL) and PI (0.5 µg/mL) for 5 min to observe the plasma membrane integrity (Celeghini et al, 2007). Then, the sample was observed under an epifluorescence microscope (NIKON - Eclipse TE300, Melville, NY, USA) at 400× magnification. All samples were evaluated within 30 min.

The PI binds to the DNA of cells with damage in the plasma membrane (Graham et al., 1990) and stains the nucleus red, while the Hoechst binds to the DNA of all cells and stains the nucleus blue (Casey et al., 1993). Four replicates of four different bulls, we analyzed with at least 200 cells counted (n=3200). The sperm membrane was classified as intact or damaged for each cell.

### Assessment of sperm capacitation by fluorescent chlortetracycline assay (CTC)

The sperm capacitation was assessed by chlortetracycline hydrochloride (CTC) modified by Cormier et al. (1997). The CTC stock solution (0.75 mM CTC, 20 mM Tris-base, and 5 mM DL-cysteine) was prepared on the day of use. We added 15 µL of the final CTC solution [300 µL of CTC stock solution mixed with 10 µL of paraformaldehyde (4%) in 20 mM Tris base and 60 µL of octane diazabicyclo - DABCO (Invitrogen Molecular Probe, Eugene, OR, USA)] to 15 µL of sperm (25 × 10^6^ spermatozoa/mL) followed by 15 min incubation at room temperature. The sample was observed under an epifluorescence microscope (NIKON - Eclipse TE300, Melville, NY, USA; 40× - excitation 440 nm and emission at 470 nm). All samples were evaluated within 30 min.

Four replicates of each bull were analyzed with at least 200 cells counted (n=3200) and classified into 3 groups, as described by Fraser (1995): F (fluorescent), intact and non-capacitated sperm with entire fluorescing surface, C (capacitated) fluorescence loss in post-acrosomal region, and RA, sperm with reacted acrosome showing fluorescence loss in post-acrosome and acrosome region with fluorescence exclusively in the middle part and equatorial region of the head.

## Assessment of the quality of sperm capacitation by the in vitro production of blastocysts

### 

#### Selection and in vitro maturation of oocytes

Oocytes were selected with grades 1 and 2 (de Loos et al., 1989) from ovaries obtained from local abattoirs. These ovaries were transported to the laboratory within 1 hour in thermo bottles containing 0.9% NaCl sterile saline at 38.5ºC and antibiotics (100 IU/mL penicillin and 100 µg/mL streptomycin). After being selected, the oocytes were washed 3 to 4 times in the Progest wash medium (Progest Biotecnologia em Reprodução Animal, Botucatu, SP, Brazil) and then transferred to the maturation plate.

The medium used for *in vitro* maturation was Progest *in vitro* Maturation (Progest Biotecnologia em Reprodução Animal, Botucatu, SP, Brazil). Maturation was carried out in Petri dishes (35x10mm, Corning Inc. Acton, MA, USA) with droplets of 100 µL immersed in mineral oil (1 oocyte/5 µL of medium) in an incubator at 38.5°C under an atmosphere of 5% CO_2_ for 22 hours.

#### In Vitro Fertilization (IVF)

Sperm selected by mini Percoll were washed for 3 min (150 × *g*) in TALP Chamberland, and the sperm concentration was determined in a Neubauer chamber to detect the fertilization dose used for IVF (2 × 10^6^ spermatozoa/mL).

The sperm were added to IVF drops of 50 µL, with one plate being used for each treatment, where they remained co-incubated with oocytes for 18 hours under the same conditions of IVM in medium Progest *in vitro* Fertilization (Progest Biotecnologia em Reprodução Animal, Botucatu, SP, Brazil).

#### In Vitro Culture (IVC)

The IVC medium used was Progest *in vitro* Culture (Progest Biotecnologia em Reprodução Animal, Botucatu, SP, Brazil). After 20 hours, the presumptive zygotes were submitted to consecutive pipetting for partial removal of the cumulus cells and spermatozoa. After that, the zygotes were transferred to drops of IVC (1 zygote/5 µL of medium) and remained incubated for 7 days at 38.5°C with a humidified atmosphere of 95% air and 5% CO_2_.

After 72 hours of cultivation, it was carried out a replacement of 60% of the culture medium (feeding). The cleavage rate was assessed 48 hours after IVF, which were considered cleaved embryos that had two or more cells without signs of fragmentation or cell degeneration. At day 7 (D7), we observed the blastocyst rate according to the criteria recommended by the International Embryo Transfer Society (Robertson and Nelson, 1998).

#### Statistical analysis

Data were subjected to analysis of variance (ANOVA) to determine the effects of treatments in the studied characteristics and the averages were compared by Tukey test at 5% probability (SAS, 2011).

## Results

### Quantitative evaluation of sperm capacitation by fluorescent chlortetracycline assay (CTC)

The percentage of non-capacitated sperm was higher (P<0.05) in the Control 0 hour (68.4 ± 5.1%) when compared to the capacitation with L-arg (45.8 ± 4.3%), Hep (25.8 ± 3.4%) and L-arg + Hep (19.2 ± 1.5%). Treatment with L-arg + Hep in the absence of COC showed the highest percentage of capacitated sperm (61.2 ± 1.2%), differing from treatments with Hep, L-arg, and the Control 0 hour (47.0 ± 4.0%, 26.2 ± 3.8%, and 18.2 ± 4.5%, respectively) (P<0.05).

**Figure 1:**
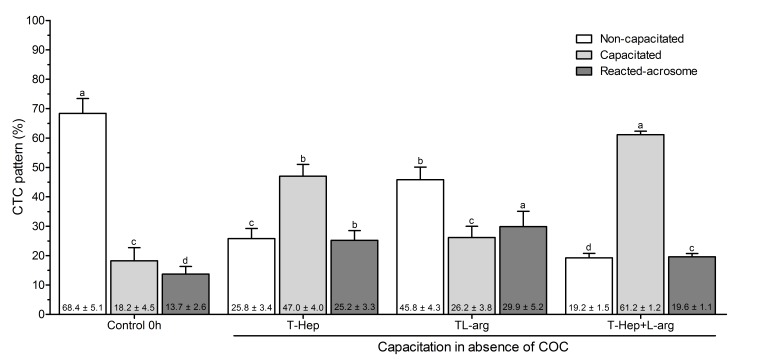
Percentage of non-capacitated sperm, capacitated, and acrosome reacted in treatments in the absence of COCs: Control 0hour, 30 min capacitation with heparin, 30 min capacitation with L-arginine, and 30 min capacitation with L-arginine + heparin. Data are presented as mean ± CI (confidence interval, P<0.05) of four repetitions from four bulls (n=3200 sperm). Different letters indicate statistical differences between treatments for the studied pattern according to the Tukey test. In each repetition, 200 sperm cells were counted. The numbers in bold at the bottom of each bar represent the mean observed in each treatment.

The percentage of sperm with reacted acrosome (RA) was lower (P<0.05) in the Control 0 hour (13.7 ± 2.6%), followed by treatment with L-arg + Hep (19.6 ± 1.1%), Hep (25.2 ± 3.3%) and L-arg (29.9 ± 5.2%) in the absence of COC. These results are summarized in Figure 1.

### Assessment of plasma membrane integrity

The statistical analysis showed no statistically significant differences in the percentage of spermatozoa with intact plasma membrane between treatments after 30 min of capacitation (Control 0 hour: 66.8 ± 7.0%; T-Hep: 67.5 ± 5.1%; TL-arg: 67.2 ± 6.5%; TL-arg + Hep: 67.4 ± 6.4%).

### Assessment of the quality of sperm capacitation by the in vitro blastocysts production

There was no statistically significant difference between the percentage of cleaved embryos of Control IVF when the sperm were capacitated with heparin in the presence of COC (78.0 ± 3.2%) and the treatments that were capacitated in the absence of COC with L-arg + Hep (82.2 ± 3.5%) or Hep (79.3 ± 4.2%, P>0.05). However, these groups differed from the group with L-arg in the absence of COC (64.4 ± 7.3%, P<0.05).

**Figure 2:**
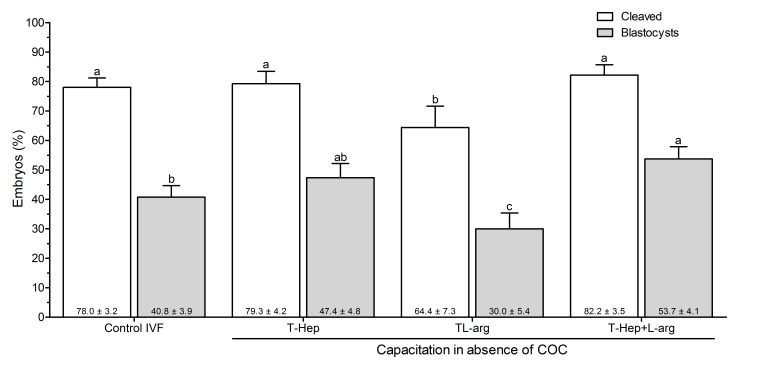
Percentage of cleaved embryos and blastocysts in the following treatments: capacitation with Hep in the presence of COCs (Control IVF), capacitation with Hep in the absence of COCs, capacitation with L-arg in absence COC, and capacitation with L-arg + Hep in the absence of COCs. Data are presented as mean ± CI (confidence interval P<0.05) of six replicates from four bulls (24 IVF trials, n=2340 COCs). Different letters indicate statistical differences between treatments for the developmental stage according to the Tukey test (P<0.05). The numbers in bold at the bottom of each bar represent the mean observed in each treatment.

The blastocyst production of the group with L-arg + Hep in the absence of COC (53.7 ± 4.1%) was higher when compared to the group capacitated with heparin in the Control IVF (40.8 ± 3.9%, P<0.05). Additionally, we did not observe differences between the group with L-arg + Hep in the absence of COC and group heparin (47.4 ± 4.8%). The group capacitated with L-arg showed the lowest blastocyst rate (30.0 ± 5.4%), different from the other treatments (P<0.05). These results are summarized in Figure 2.

## Discussion

In cattle, nitric oxide plays a fundamental role in the sperm capacitation process (O’Flaherty et al., 2004; Rodriguez et al., 2005; Leal et al., 2009). Although the sperm can synthesize NO during capacitation, recent studies demonstrate that exogenous increase of NO from the inclusion of L-arg to the capacitation medium improves sperm quality in a dose-dependent manner during incubation (Leal et al., 2009; Jagan Mohanarao and Atreja, 2012). The availability of L-arg for NO synthesis is determined *in vitro* mainly by L-arg concentration in the culture medium used (Ignarro, 2000). Moreover, *in vivo,* the capacitation process occurs throughout the female reproductive tract, especially in the oviduct, where a sperm reservoir is formed (Hung and Suarez, 2012). In the same way, the primary source of NO production for the sperm capacitation *in vivo* originates from the female reproductive tract (Lefièvre et al., 2007), being concordant with the improvement observed by the addition of L-arg to the capacitating medium. Although the NO dosage has not been performed in our research, many studies demonstrate the NO synthesis by cattle sperm using L-arg as a substrate during the *in vitro* capacitation (Leal et al., 2009; 2012; Santana et al., 2016).

Here we used 1 mM L-arg in the capacitation medium in absence of COC for 30 min, as we previously showed for improving sperm capacitation (Leal et al., 2012).We observed that the inclusion of L-arg to the capacitation medium containing heparin increased the percentage of capacitated sperm and kept down the percentage of sperm that presented acrosome reaction and that was non-capacitated. The results of this study are consistent with those we reported (Leal et al., 2012) at 4 hours of capacitation.

Spermatozoa with a high percentage of acrosome reaction at the time of insemination showed low fertility (De Jonge and Barratt, 2017) that may result in a decrease in the cleavage rate. Thus, the increase in the percentage of non-capacitated and acrosome-reacted sperm in L-arg group may have contributed to the decline in the fertilizing potential of the sperm, and consequently on the rate of the *in vitro* production of blastocysts.

During the sperm capacitation of mammals, NO acts in the cAMP and the extracellular-regulated kinase (ERK) signaling pathways (Gangwar and Atreja, 2015). Heparin primarily acts in the membrane (Parrish, 2014), which facilitates the entry of ions, especially calcium. Ca^2+^ intake leads to an increase in the intracellular pH and consequently hyperactivation (Parrish, 2014; Gangwar and Atreja, 2015). The increase of Ca^2+^ and NO concentrations activates the soluble adenylate cyclase (sAC), stimulating the cAMP synthesis and protein tyrosine phosphorylation through protein kinase A (PKA). As reviewed by Gangwar and Atreja (2015), the extracellular-regulated kinase (ERK) plays a role in the sperm capacitation, motility, and acrosome reaction. In the intracellular environment, ERK is activated by NO, leading to protein tyrosine phosphorylation (Gangwar and Atreja, 2015). Thus, L-arg may stimulate the ERK pathway and potentialize the cAMP pathway. More studies are needed to address this hypothesis in bovine.

In this study, no difference in sperm was observed between Control 0 hour and the groups treated in the absence of COC for 30 min, which showed intact membrane. It is worth noting that the average of sperm that had intact membrane was about 67%, similar to the percentage of capacitated sperm in the presence of L-arg + Hep (61%). This observation suggests that almost all the sperm that presented plasma membrane integrity were eligible for *in vitro* capacitation. Since ionic events, such as changes in intracellular concentration of Ca^2+^, H^+^ (Pons-Rejraji et al., 2009), and HCO_3_^-^ (Breininger et al., 2010) modulate the capacitation, only spermatozoa with plasma and acrosomal membrane integrity can be capacitated.

On *in vitro* embryo production, no difference was observed in the cleavage rate between treatments with heparin in the absence of COC comparing to Control IVF, corroborating with the results obtained by Santana et al. (2016) and Leal et al. (2012). The group treated with L-arg demonstrated the lowest cleavage rate, as well as the lowest rate of capacitation and *in vitro* production of embryos. These data corroborate with those obtained by Paes de Carvalho et al. (2003), which demonstrate that, in the presence of 10^-9^ M sodium nitroprusside (SNP, NO donor), the penetration rate of oocytes by capacitated spermatozoa is reduced when spermatozoa are treated with heparin. Santana et al. (2016) found no additive beneficial effect of these capacitation agents, although they have been tested on the capacitation in the presence of COCs (traditional method). Therefore, in this method used by the authors, the L-arg could be acting both on sperm (Leal et al., 2009) and COCs (Dubeibe et al., 2017), and thus this result indicate that the observed effect was not exclusively by the action of L-arg in sperm capacitation.

The increase in the blastocyst rate in the capacitation group with L-arg + Hep in the absence of COCs in comparison with Control IVF and T-Hep may occur due to the greater number of capacitated sperm in addition to the reduced level of acrosome-reacted sperm. Prior to fertilization *in vitro*, the sperm cells (Leal et al., 2009, 2012) and the COCs (Viana et al., 2007; Matta et al., 2009) synthesize NO, similarly as in the oviduct (Lefièvre et al., 2007). Moreover, the female reproductive tract also produces heparin (Fukui et al., 1990), also relevant to the capacitation process. In this study, we demonstrate that the treatment with L-arg + Hep could mimic the female reproductive environment *in vitro*. With this reasoning, we previously demonstrated that using L-arg in addition of heparin in the capacitation media 41 proteins were differentially abundant compared to control (only with heparin). Many of these proteins being associated with key roles in sperm capacitation, fertilization and embryonic development (Maciel et al., 2018).

The L-arg + Hep capacitation method performed in the absence of the COC has proven effective in the evaluation of different treatments on the capacitation. This observation indicates that the oocytes were not influenced by L-arg and heparin during the fertilization process due to removal of treatments after 30 min.

Thus, our findings indicate that the addition of L-arg to capacitation medium containing heparin increases the number of *in vitro* capacitated sperm and decreases the number of non-capacitated and acrosome-reacted sperm with 30 min of culture in the absence of COCs. Moreover, these experiments have shown no interference on the percentage of sperm showing plasma membrane intact. Furthermore, the addition of L-arg in the capacitation medium with heparin in the absence of COCs was the most efficient method of blastocysts *in vitro* production in cattle. Since this pre-incubation method in absence of COC was the most effective, we further emphasize that it could be used to assess the role of other substances in the sperm capacitation and its effect on IVP.
